# Wearable Sensors with Internet of Things (IoT) and Vocabulary-Based Acoustic Signal Processing for Monitoring Children's Health

**DOI:** 10.1155/2022/9737511

**Published:** 2022-04-28

**Authors:** Kapil Kumar Nagwanshi, Ajit Noonia, Shivam Tiwari, Nitika Vats Doohan, Vijeta Kumawat, Tariq Ahamed Ahanger, Enoch Tetteh Amoatey

**Affiliations:** ^1^Department of Computer Science and Engineering, ASET, Amity University Rajasthan, Jaipur, India; ^2^School of Computing & Information Technology, Manipal University Jaipur, Jaipur, Rajasthan 303007, India; ^3^Department of Computer Science and Engineering, G L Bajaj Institute of Technology and Management, Greater Noida, Uttar Pradesh, India; ^4^Department of Computer Science and Engineering, Medi-Caps University, Indore, Madhya Pradesh, India; ^5^Department of Computer Science and Engineering, Jaipur Engineering College and Research Centre, Jaipur, Rajasthan, India; ^6^College of Computer Engineering and Sciences, Prince Sattam Bin Abdulaziz University, AI-Kharj, Saudi Arabia; ^7^School of Engineering, University for Development Studies, Tamale, Ghana

## Abstract

The brain is the most complex organ in the human body, and it is also the most complex organ in the whole biological system, making it the most complex organ on the planet. According to the findings of current studies, modern study that properly characterises the EEG data signal provides a clear classification accuracy of human activities which is distinct from previous research. Various brain wave patterns related to common activities such as sleeping, reading, and watching a movie may be found in the Electroencephalography (EEG) data that has been collected. As a consequence of these activities, we accumulate numerous sorts of emotion signals in our brains, including the Delta, Theta, and Alpha bands. These bands will provide different types of emotion signals in our brain as a result of these activities. As a consequence of the nonstationary nature of EEG recordings, time-frequency-domain techniques, on the other hand, are more likely to provide good findings. The ability to identify different neural rhythm scales using time-frequency representation has also been shown to be a legitimate EEG marker; this ability has also been demonstrated to be a powerful tool for investigating small-scale neural brain oscillations. This paper presents the first time that a frequency analysis of EEG dynamics has been undertaken. An augmenting decomposition consisting of the “Versatile Inspiring Wavelet Transform” and the “Adaptive Wavelet Transform” is used in conjunction with the EEG rhythms that were gathered to provide adequate temporal and spectral resolutions. Children's wearable sensors are being used to collect data from a number of sources, including the Internet. The signal is conveyed over the Internet of Things (IoT). Specifically, the suggested approach is assessed on two EEG datasets, one of which was obtained in a noisy (i.e., nonshielded) environment and the other was recorded in a shielded environment. The results illustrate the resilience of the proposed training strategy. Therefore, our method contributes to the identification of specific brain activity in children who are taking part in the research as a result of their participation. On the basis of several parameters such as filtering response, accuracy, precision, recall, and F-measure, the MATLAB simulation software was used to evaluate the performance of the proposed system.

## 1. Introduction

In the last few decades, nonlinear dynamical analysis has developed as a unique tool for the study of complex systems, which has the potential to revolutionise the field. To better understand the dynamics of the complicated underlying behaviour, the nonlinear analysis approach may be used in EEG data with success [1]. The noninvasive character of EEG has played a significant role in its development as a tool for mental health assessment. Using the concepts of nonlinear dynamics and deterministic chaos, the technique entails the identification of system attractors and their invariant characteristics, as well as the characterisation of the system attractors. When compared to standard linear approaches such as Fourier transforms and power spectrum analysis, this method is considerably better in terms of performance [2]. A particularly significant study field of medicine is the analysis of nonlinearity in electroencephalograms (EEGs), which has therapeutic implications as well as research into brain dynamics, in light of the fact that our thesis is primarily concerned with higher order statistics using surrogate data for sleep EEG analysis [3].

The relevance of biological time-series analysis, which shows generally complicated dynamics, has long been recognised in the field of nonlinear analysis, and this is particularly true in the field of cancer research [4]. Due to the exceedingly irregular and nonlinear structure of these biological systems, it is difficult to perform a complete analysis of them. Because of their foundation in the notion of chaos, nonlinear dynamical methods have been applied in a range of sectors, including medicine and biology, to considerable success. Nonlinear metrics such as correlation dimension for diseased systems have been determined by various methods, and it has been demonstrated that they are valuable indicators of pathologies in some cases [5].

The theory of nonlinear dynamics provides us a new window for understanding the behaviour of the electroencephalogram.

### 1.1. Brain Signal Detection Methods

Different techniques for recording electrical or magnetic activity, as well as functional magnetic resonance imaging (fMRI), magnetoencephalography (MEG), functional near-infrared (FNIR) imaging, and positron emission tomography (PET), may be used to identify and evaluate brain signals (PET). However, because of the complexity of the technological requirements, the high cost, and the lack of real-time capabilities, MEG, fMRI, and PET are not suited for routine usage at this time [6]. In the near future, only electrical field recording and FNIR are expected to be of practical relevance in clinical settings, according to the experts. Electrocorticography (ECOG) [7] is a technique for recording electrical activity in the brain at several locations including the scalp, cortical surface, and inside the brain (Local Field Potentials/LFP or spike train). Each strategy has its own set of benefits and drawbacks to consider. Local Field Potential (LFP) [8] techniques such as ECOG offer strong topographical resolution and can operate across a large frequency range. When employing intrusive signal techniques to capture intercortical neural events in primates, brain-computer interfaces (BCIs) [9] have shown significant promise in direct brain control of external devices, such as the ability to restore self-feeding by regulation [10]. But they are intrusive, requiring the implantation of electrodes on or inside the cortex to get results. The following are the primary problems of invasive BCIs which must be addressed before they may be used in therapeutic settings: long-term safety and signal stability and duration. As a result of its lower clinical threshold and simplicity of application, the noninvasive EEG approach has been widely investigated. Electroencephalography (EEG) recordings, on the other hand, are occasionally sensitive to contamination by electromyography (EMG) or electrooculography (EOG) activity from cranial muscles [11].

Noninvasive approaches, in contrast to invasive methods, have an exceptionally low signal-to-noise (s/n) ratio, which presents a significant obstacle in the development of EEG-based brain-computer interfaces. Traditional methods of improving the s/n ratio include repeated averaging, which may be used to create Event Related Potentials (ERPs), which can be acquired by averaging over trials that are time-locked to the stimulus [12]. However, because of the demand for several measurements, the transmission speed is significantly lowered [13]. Users may be taught to manage their brain activity, such as by modulating the Slow-Cortical Potentials (SCPs) or the 8–12 Hz sensorimotor Mu rhythm, as an alternate technique of improving the s/n ratio for reliable BCI control. The s/n ratio will rise as a consequence of individuals learning to successfully manage their brain activity. Once people learn to effectively regulate their brain activity, it is predicted that the variation in their EEG signal would decrease [14]. For SCPs or sensorimotor Mu rhythms, short-term training can be useful, but long-term training is usually necessary, since spontaneous EEG activity is so erratic [15].

**Figure 1:**
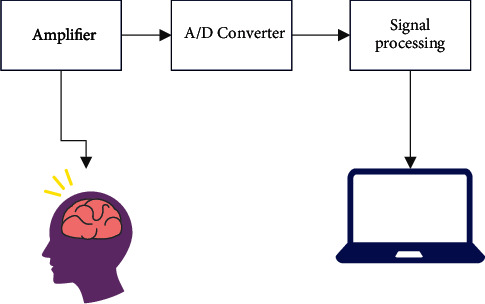
Process of EEG signal.

Figure 1 shows the process of EEG signal.

The electroencephalogram (EEG) is produced by the simultaneous firing of 10000–100000 neurons and may be measured as a voltage differential (voltage) via placing two electrodes on the head.

It is necessary to activate a region of cortex underneath each electrode which is roughly 5 cm^2^ in size in order to measure the potential. It has been shown [16] that since the EEG can accurately represent all the activity of the brain, it is a highly effective instrument in the area of clinical neurophysiology. Electroencephalography (EEG) is a noninvasive instrument that may be used for a variety of purposes, including (i) comprehending the dynamic complex functioning of the brain, (ii) monitoring its various physiological states, and (iii) diagnosing neurological illnesses. In order to collect EEG data, which are noninvasive electrical brain impulses, electrodes are put on the scalp and connected to a recording device (sometimes in form of a cap) [17]. The electrodes are cup-shaped and are implanted at certain points on the scalp to get the desired results. In these electrodes, the skin never comes into direct contact with the electrode material. EEG gel or paste serves as an interface medium between the electrode and the skin during the recording process. It is necessary for the electrodes to provide sufficient capacity to hold an electrolyte and collect the electrical signal [18]. The electrode-skin interface impedance is dependent on the thickness of the interface layer, the area of the electrode's surface, and the temperature of the electrolyte at the time of measurement. Skin, electrolyte, and electrode are shown in Figure 1 as the electrical equivalents of the three components. Resistance and capacitive components make up the electrode-tissue contact. The ions form parallel plates because of the interaction between the metallic electrode and the electrolyte [19].

According to the findings, there are major differences in brain wave patterns for various everyday tasks. The EEG signal is primarily used for the investigation of the changing electric potential associated with human activities. The brain, as it is also called, is one of the most complicated structures in the whole biological system [20].

The rest of the paper is organized as follows: Section 2 presents background analysis, Section 3 presents the proposed methodology, and Section 4 discusses the experimental results.

## 2. Background Analysis

According to the findings of this research, Ding et al. [21] used scalp EEG data for primary interpretation. Deep learning approaches have been utilised, and they have been compared to more classic linear methods. Accuracy of decoding was restricted in experiments that used EEG because of contamination from artefacts and because there was insufficient precise information retrieved from the scalp to recreate complicated motions. Although high signal resolution gained by intrusive procedures may be essential in order to comprehend movement control, with the development of modern signal processing techniques as well as quick computing and machine learning technologies, this may no longer be necessary. In Aziz et al.'s work [22], the electroencephalogram (EEG) is a highly valuable tool for understanding the neurological dysfunction caused by stroke, as well as for improving therapy and rehabilitation. The Fourier Transform is the foundation of the majority of the available approaches for diagnosing stroke from the EEG data, which are described here (FT). When measuring symmetrical blood flow between the left and right cerebral hemispheres, the Brain Symmetry Index (BSI) uses Fast Fourier Transform (FFT) coefficients, for example. Between zero and one, the symmetry index is measured, with one reflecting the highest imbalance in blood flow [23]. It is well known that the Traditional Fourier Transform (TFT) has limitations in the study of nonlinear and nonstationary signals. The present BSI and its variants, as well as their derivatives, may be affected by these transformation features. To define the BSI-HHT, this work proposes a BSI based on the Hilbert-Huang Transformation (HHT). When a signal is broken down into Intrinsic Mode Functions (IMFs) with a trend, high-speed tracking (HHT) may be used to retrieve instantaneous frequency data. Instead of the FFT, the HHT coefficients will be utilised in the computation of the BSI index [24]. In this paper, an experiment to verify the performance of the BSI-HHT approach is carried out in order to compare it to the already available BSI technique. Specifically, the EEG signals from participants with Middle Cerebral Artery (MCA) disease and healthy subjects were employed in this work. It is more accurate to interpret the data generated using the suggested BSI-HHT method because it corresponds to the stimulation technique on the data gathered, particularly at a certain frequency band [25]. The HHT coefficient may also capture the nonstationary and nonlinear behaviour of the electrode of interest, which can be determined by examination of the data. In the work of Al-Fahoum and AL-Fraihat [26], generally speaking, the purpose of the review is to shed light on how the features of the EEG signal are extracted and to demonstrate how rapidly the technique used for signal extraction and the reliability of its characteristics may be determined. Furthermore, how these extracted characteristics would convey distinct mental states for different mental activities and would be able to create an appropriate categorization and translation of mental tasks was investigated and found to be promising [25]. In order to avoid losing critical information at an inopportune time, the speed and precision of the step of extracting the features of the EEG signal processing are very significant. As discussed so far in the literature reviewed, the wavelet method is a solution for instabilities in signals which is comprised of the representation of wavelets, which are a group of functions derived from the mother wavelet by means of processes of expansion and translation, as well as the representation of wavelets [27]. The variable size window is the most important parameter of this approach, since it ensures that the proper time-frequency resolution is maintained over the whole frequency range of operation.

Cranstoun et al. [28] provided a novel time-frequency spectrum estimate approach for multichannel data that is applicable to epileptic form Electroencephalography (EEG). Smooth Localised Complex Exponential (SLEX) functions are used to implement the approach, which are time-frequency localised versions of the Fourier functions. As a result, they are particularly well suited for the analysis of no stationary signals whose spectral features change with time. The SLEX functions are orthogonal and localised in both time and frequency at the same time because they are generated by applying a projection operator rather than a window or taper to the input signals [29].

In the field of digital image processing, picture enhancement is one of the most straightforward and exciting areas to work in. The purpose is to draw attention to particular aspects of a picture or to draw attention to certain characteristics of interest (Hames et al. [30]). If the brightness of bone or brain tissue in the input picture is reduced or increased, this may help to enhance the quality of the distorted image [31]. The dualistic subimage histogram equalisation approach is used in this improvement technique. A segmentation strategy has been developed based on directional homogeneity and using a modified measure. Uniformity concerning the two seed templates oriented in opposing directions is required for this method to work. The search for pixels is restricted to a limited number of directions. Only eight directions are taken into consideration, resulting in quick and reliable extraction of brain picture pixels. The picture parts are compared to the templates using a method that requires less computing efficiency [32].

Among those who have contributed to this work are Siuly et al. [33] In the mining business, a wearable helmet for humans may be an extremely important instrument for monitoring the health of workers. However, there has been minimal investigation into the identification of human emotions in harsh environments [34].

The fusion method for stress level has a correct subsequent function to the negative emotional shift, [35] which is another benefit of using this algorithm [36]. The amount of anxiety experienced by a person may be quantified using this approach. Thus, improving operational safety and avoiding inappropriate miner operation may be accomplished by the use of this strategy [37].

It has been discovered that an EEG-based Brain-Computer Interface for treatments is inadequate for restoring upper-limb function after a stroke that has impacted the brain. The BCI is controlled by brainwaves in the gamma band (8–15 Hz) (I) Delta FFT findings consist of EEG, data, and five frequency bands between them (0.5–4 Hz), (b) theta (4–8 Hz), (a) alpha (8–13 Hz), (b) beta (13–30 Hz), and (c) gamma (50–60 Hz) [38], respectively (30–50 Hz). When compared to rest, imagined activity reduces the spectral power in this region by up to 100 dB, which is significant. The original BCI computed the maximum or mean of the mu band in order to identify this difference; however, they may not be the most accurate functions. Using a combination of time-domain and frequency-domain approaches, it is possible to evaluate physiological data while overcoming numerous restrictions, such as lower accuracy owing to Fourier phase suppression. The enormous computational complexity that standard signal analysis algorithms must contend with and the ultimate exploration of the clinical significance and discriminating capacity of higher-order statistics in the context of emotional state.

Originally, the EEG decoding pipeline relied on a single data point to determine whether or not the individual was resting or envisioning movement: either the mean or maximum of the mu-band spectrum, depending on which seemed to provide the most accurate findings. Scientists may now better comprehend the role of Theta wavelength synchronisation thanks to the study, which has provided them with a framework for understanding how their data connects with real activity. In order to develop BCIs, it is necessary to understand and classify the signals coming from the brain, which may be accomplished using suggested Resilient Direct Neural Network (RDNN) and the Learning Neural Network Classification (LNNC) methodologies.

## 3. Proposed Methodology

**Figure 2:**
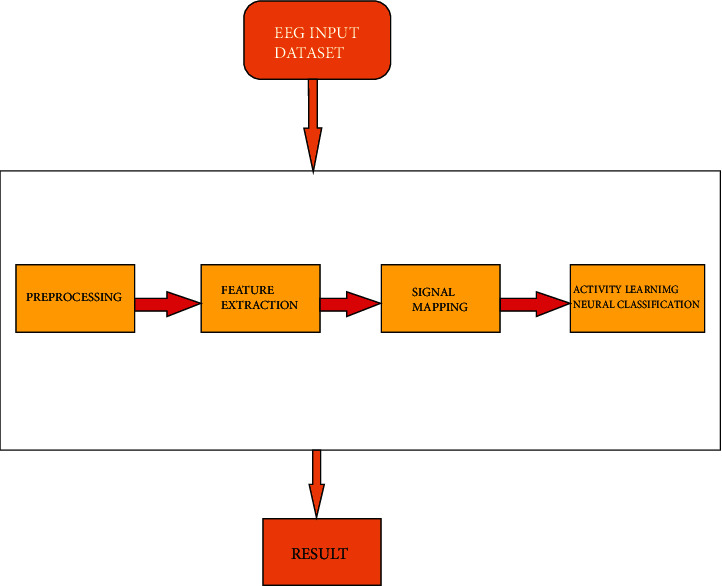
Proposed system's block diagram.

Figure 2 depicts the wavelet transform and the Learning Neural Network Classification (LNNC) algorithm, both of which are based on human activity watching. To be more detailed, the suggested framework is divided into four stages. Preprocessing, feature extraction, user profile mapping, and categorisation are all aspects of data processing. Each of the phases includes present tasks that must be completed upon detection of a human brain activity employing an EEG signal in each of the stages.

### 3.1. Materials and Method

On the basis of human activity observation, the continuous wavelet transform and the Learning Neural Network Classification (LNNC) approach are shown in Figure 2. Preprocessing, feature extraction, user profile mapping, and classification are the four steps of the proposed framework, with preprocessing being the first.

### 3.2. Input Acquisition of Proposed Work

**Figure 3:**
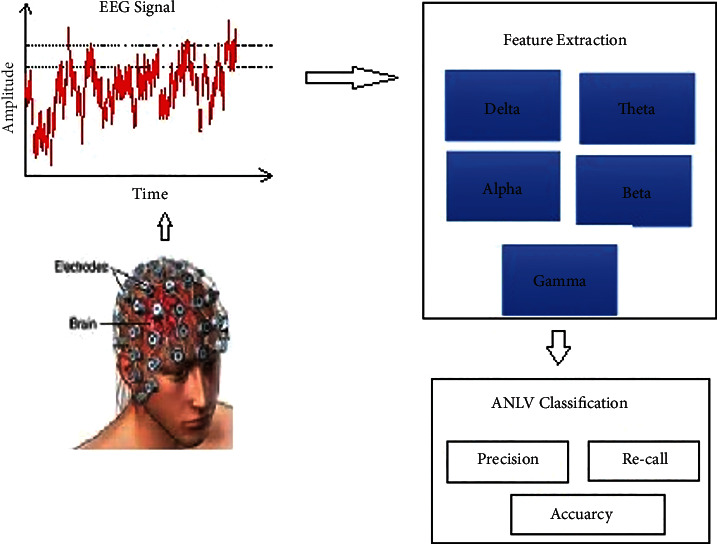
Signal recognition procedure.

The signal recognition capabilities of the proposed system are explained in further detail in the next section.  Figure 3 depicts the signal pattern identification procedure as it is carried out step by step.

A total of 13 out of 27 channels were utilised by the best person who was identified using the GA-SVM approach applied to the NIPS dataset.

When compared to the all-channel selection, the GA-SVM significantly improved the average classification accuracy on a per-channel basis.

The wrapper method improved overall classification accuracy by 3.15 percent, bringing it to 0.8527 percent.

The EEG signal is collected from the human scalp as shown in the preceding picture, and, after identification of the EEG signal, feature like values are extracted from the signal for the purpose of computing and determining classification accuracy as shown in the following figure.

A binary string of this kind might be regarded to constitute the genetic information of a single person. Initialization of the population begins with a random selection of several people from which to build the population. Simply said, the features of such a person are fully dictated by the thread that binds them together. Every person is given a fitness value, which is determined by creating and categorising the associated dataset for that individual. For this reason, only chosen channels (as determined by the ones in the text) have their time series concatenated, with the “0” channels being excluded. The collected dataset is then utilised to train SVMs using a neural network. In order to determine an individual's fitness, the accuracy of classification is measured.

### 3.3. Adaptive Wavelet Transform: Preprocessing

The filter section of the preprocessing step is responsible for filtering out the noisy values from the incoming EEG signal. A wavelet filter is used in the preprocessing signals, [39] which retains the low-frequency component to make inspection more convenient and straightforward. It is composed of a few subgroups such as delta and other related ticks, such as arrhythmic and paroxysmal, which make up the low-frequency signal. Each of the highlights relates to the different activity of the brain neurons when subjected to distinct electric stimulation patterns. The channel incidence is within the range of 4 Hz and corresponds to the usual musicalness of children and adults alike. It is theta frequency, which is in the range of 4 to 7 Hz [40], which indicates moderate brain activity, which is normal in children but aberrant in adults. In the 8 to 12 Hz range, there is an alpha frequency that manifests itself when the eyes are closed and when they are opened, demonstrating the casual brain status. The beta waves are the irregular beats of the brain that have a frequency greater than 13 Hz [37] and occur at a higher frequency than the rest of the brain. Arrhythmic and paroxysmal highlights are also eliminated from the EEG waveform, as is any other kind of abnormality.

The information signal is composed of many components, whose signal amplitudes vary. These components are denoted by the letters a, b, c, and d. In certain cases, the signal's amplitude is low; therefore, we increase it in order to identify ourselves throughout the extraction process of the features. Multilevel observation is used in conjunction with an adaptive wavelet processing to generate the signal, which results in a waveform with improved character. While the waveform is at a positive level, each part of the waveform must have different values to be considered. As a starting point, the default settings for each of the EEG signals and signals and their involvement in this system are as follows:

(1) $$\begin{array}{c} W\left(Z\right)W\left(Z^{-1}\right)+W\left(-Z\right)W\left(-Z^{-1}\right)=1,\\ H\left(Z\right)=ZG\left(-Z^{-1}\right). \end{array}$$

An order of the filter increases distance (index can be obtained):

(2) $$\begin{array}{c} Wi+1\left(Z\right)\;=\;W\left(-Z^{-2i}\right)Wi\left(Z\right)\;Hi+1\left(Z\right)\;\\ =\;H\left(-Z^{-2i}\right)Hi\left(Z\right). \end{array}$$

1. Begin
2. Set the standard plan P to its initial state.
3. Examine the input label IP.
4. For distinctly standard P at time Ti from IP, calculate the following:
5. P is an abbreviation for Adaptive Wavelet Transform (Pi). If Pi is greater than or equal to minTh, then P is added to the signal pattern P, which is *P* = (*P* + Pi).
6. End
7. Stop

### 3.4. Feature Extraction

The performance of classification is also affected by the selection of a subset of all available EEG channels from which to train. This selection is usually made before any features are calculated, but, in a broader sense, the selection of channels can be considered a feature selection in and of itself. On the other hand, the selection of acceptable channels is usually either medically justified or determined based on the statistics of example data, for example, using the approach of common spatial patterns. For this spatial filtering of EEG data, multiple variations of independent component analysis have been effectively employed for a variety of applications.

Feature vectors are created and the actual classification issue is performed using techniques such as linear or kernel discriminant analysis artificial neural networks, decision trees, and other algorithms once the feature vectors are calculated.

A genetic algorithm (GA) is used in this paper to select the most promising EEG channels for classification using support vector machines. The approach is described in detail in the paper (SVM). The data utilised in our studies will be described in depth in the next part, after which the GA-SVM approach will be detailed. The findings of this strategy are then compared to those obtained via physiologically driven feature selection methods and, if appropriate, to those obtained by brute-force channel selection.

It is necessary to fragment the input signal into a number of subgroups before using the wavelet inspection approach. The appropriate ripple and the number of degradation heights are used to disrupt down the signal and extract useful information from it. The stages of degradation level are selected in accordance with the prevalent frequency. The levels are chosen in such a way that those parts of the signal that match closely to the frequencies required for categorization are preserved. Because the EEGs provide only little useful information beyond the frequency range of 30 Hz to 173.6 Hz [34], five unique groups are selected with a single estimate go.. At this point, the wavelet *t* and the number of breakdown levels *N* are determined by the user.

**Figure 4:**
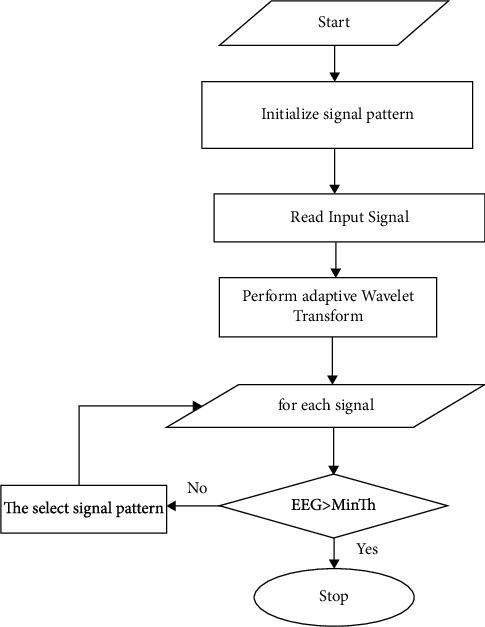
Block diagram of proposed work.

The preprocessing of the planned task is shown in Figure 4. According to (3), the energy at different degeneration levels is transmitted.

(3) $$\begin{array}{c} E=\left|dj\;,k\right|, \end{array}$$

where *j* denotes the degree of decomposition and *k* denotes the order of decomposition, both of which have a value of 4. The energy of each band is used to construct the characteristic vector “*V*,” which is constructed independently of the others. The disintegrating wave signal at every level of a band is combined with other characteristics such as a band “*Ei*,” duration “*t*,” and age “an” to create a feature vector that may be used to construct a feature vector. *V* is a feature vector that contains the following characteristics: six-band data; energy; time; omega; theta; alpha; lambda; abnormal heart; like how; and other characteristics.

Feature vector is as follows:

(4) $$\begin{array}{c} V=ф\left\{Ei,t,a,de\;lta,theta,alpha,beta,arrhythmic,paroxysmal\right\}. \end{array}$$

Each feature vector from the feature set has values denoted by the letter “*V*,” and it is also utilised to do frequency inquiry and probability calculation in addition to other tasks.

The existence of alpha, beta, delta, and theta values is determined by utilising the if/else conditional statement.

- If the frequency of energy is less than 3 Hz,
- Vi (de) is equal to one.
- Else,
- if the energy is more than 3.5 Hz and less than 7.5 Hz, Vi (th) = 1; otherwise,
- if the energy is greater than 7.5 Hz and less than 13 Hz,
- Vi (al) = 1; otherwise,
- if the energy is greater than 13 Hz,
- Vi(be) = 1;
- End

### 3.5. Signal Mapping

It is just the insights into the EEG that are contained in the deleted neuron feature, which is part of the profile informative collection that provides client profile information. When using client profile mapping, the subtle client aspects are separated from the informative collecting process for the profile information. The client's adolescent history will be reviewed, and the incidence of outline to x-beams and arcs will be determined. Moreover, the number of head injuries that have occurred to the individual is recorded. The feature extraction arrangement will provide a larger feature set because of the utilization of separate client profiles. In order to predict the likelihood of a seizure recurrence in the future, researchers examine the medical histories of the patients who participated in the study. The accompanying programme examines the client outline and tests for a competition before generating the course regards for the nervous network. The eye is as follows: The frame is held by the neuron vector.

(5) $$\begin{array}{c} \varphi \left(Vi\right)=\varphi \left(Vi\right)+\left\{RF+HF+NF+FF\right\}. \end{array}$$

For example, RF denotes the periodicity of radiation, HF indicates the presence of head traumas, NF denotes the frequency with which the compressor was used, and FF denotes the number of individuals impacted by cancer in the household.

The developed feature set is prepared for use in the construction of a neuron set. The neuron set is created with the help of the fluffy reasoning run set that has been stored in the information base. The seizure weights are calculated for each type of seizure, and the weights are assigned to each class in accordance with the number of processed neurons. The constructed system is put to use for the detection of activity.

It is possible to construct an activity learning machine with a single layer of masked centers, in which the masses associated with inputs to veiled hubs are distributed arbitrarily and never updated. Finding the weights between hidden hubs and outputs is done in a single step, which is essentially the same as learning a straight model. Guang-Bin Huang coined the term “Activity Learning Machine” (ALM) to describe these types of models. The models may give excellent hypothetical implementation and learn a significant number of times faster than systems developed through backpropagation when required.

An arrangement of straight circumstances is measured by the ALM definition up to the secret weights connecting the shrouded layer to the output layer, which is where the ALM definition prompts are measured. A pseudoreverse arrangement of Moore-Penrose summed up direct conditions is used to get the organization of this general arrangement of direct circumstances. The ALM method, as well as its feasibility for use in turbulent temporal arrangement forecasting and classification problems, is discussed in this paper.

Persistent likelihood thickness work is used to determine the characteristics of this ALM method, which are picked and settled arbitrarily. It is stated in the ELM hypothesis that each and every parameter associated with the hidden neurons is haphazardly generated by chance from the preparation tests.

(6) $$\begin{array}{c} {\left\{\left(Xk,tk\right)\right\}}^{N}. \end{array}$$

For a number of samples, where

(7) $$\begin{array}{c} Yl=\left[yl1,\;yl2\ldots \ldots ylo\right],\\ Y1=\;\left[y11,y12\ldots \ldots ylo\right]. \end{array}$$

A standard single-layer feedforward neuron with *N* neurons is calculated as

(8) $$\begin{array}{c} \sum \beta j\;\left(xjyl\;+\;cj\right)=ok,\\ 1=1\ldots .\;.N. \end{array}$$

We have that *wi* = [*wi*1, *wi*2,…, *win*]*T* is the input weight of the layers in the *i*^*tℎ*^ hidden.

*xij* represents the inner products of *xj* and *yl*. The above equation can be denoted as

(9) $$\begin{array}{c} Ig=ο, \end{array}$$

(10) $$\begin{array}{c} \begin{array}{cc} Hidded\;Layer\left(I\right) & =⟦g\left(w1xk+b1\right)\cdot \left(wNxN+bN\right)⟧NxN\left(w1xN+b1\right)\\ & \cdot g\left(wNxN+bN\right). \end{array} \end{array}$$

**Figure 5:**
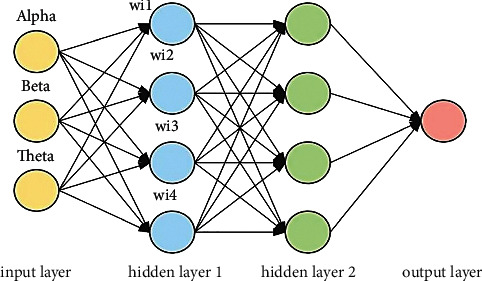
LNNC based network classification.

A neural network classification of active learning vector area has been suggested in this study for the classification of EEG signals, and it has been tested (Figure 5). The algorithm is taught to employ certain delta, gamma, gamma, gamma, arrhythmia, and paroxysmal characteristics, among others. It has been evaluated during the test data (arrhythmia, paroxysmal) collected during a training phase to determine how well the system works.

The active learning vector process is comprised of the following steps: each neuron in the neural network has an activation function that is proportional to the weight *Wji*, which is defined as

(11) $$\begin{array}{c} A\left(X,w\right)=\sum_{i=o}Wji. \end{array}$$

The sigmoid function related to the output function is shown in Figure 5.

Therefore, error function of each neuron output is

(12) $$\begin{array}{c} E\left(x,w,d\right)={\left(0j\left(x,\;w\right)-dj\right)}^{2}, \end{array}$$

where *dj* denotes the sum of the *j*th elements of the intended response vectors and the error output layer is defined as the sum of all neurons in the desired response vector.

- Input: signal type
- Class D as a result of the output.
- Start
- Region S = carry out the most extensive neuronal categorization possible.
- Preprocess signal (signal sig) = signal sig
- KSDF is an abbreviation for Activity Learning Neural Vector Classification. If KSDFTh1>, then Class A is appropriate.
- If KSDF Th2> is used, then Class B is used.
- Alternatively, if KSDF Thn>, then Class N.
- Stop.

The activity learning neural vector classification algorithm and the adaptive wavelet transform are used to observe human brain activity, and the results are presented in a report. The approach first performs adaptive wavelet transform filtering and then captures the signal for EEG feature extraction once it has completed the filtering.

## 4. Results and Discussion

Investigations have been conducted into the execution of different EEG state estimates in each of the signal processing components in order to increase the accuracy of EEG state location while maintaining or improving direct quality performance. The three alternative approaches that were offered, as well as each plan, were revised and assessed for their ability to be implemented.

The dataset that was utilised in this study is shown in Table 1. For different signal processing metrics, the productivity of the EEG estimates derived from the suggested approaches was determined by using the previously specified informative dataset. The recommended diverse separate approaches, as well as each plan, were implemented and assessed in terms of their effectiveness.

Matlab was used to update the EEG identification approach that was developed in accordance with the suggested strategy. Using wavelet analysis, both the EEG signal with wavelet inspection and the suggested method have been updated and assessed for their usefulness in estimating EEG. Tests were carried out on the desired EEG signal in order to determine the suitability of the proposed framework, and the signal was rebuilt by long-suffering the 4000 Hz inspection amount. The outcomes of the approach are exposed in this section.

**Figure 6:**
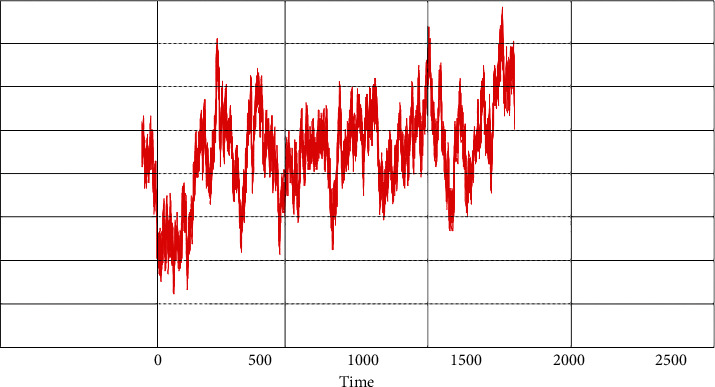
Actual EEG communication of subject-1.

Figure 6 shows the real input EEG communication during child active phase (subject-1).

**Figure 7:**
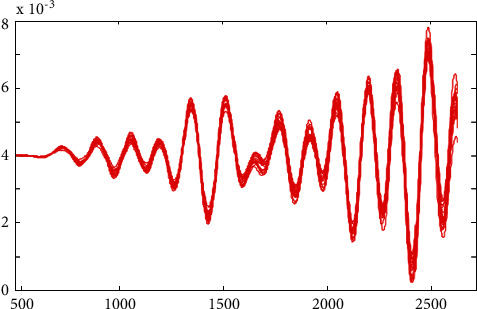
Simulated tired detected waveform for theta band.

**Figure 8:**
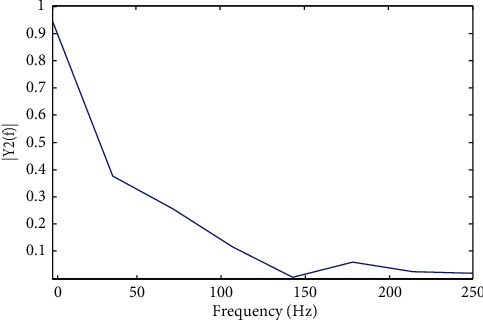
Amplitude power spectrum for theta band.

Figure 7 depicts the waveform of the detected EEG fatigued from the input waveform, which was obtained from the input waveform.  Figure 8 illustrates that the waveform recognised by EEG and replicated for the theta band from replication is successfully completed.

The implementation of the various classification units is shown in Table 2. Analyses of the measurements are carried out in accordance with the contribution and treatment of both EEG and ECG signals in order to produce genuine positives or false negatives in the database for each measurement.

Examination in Table 2 shows that the proposed strategies, such as LNNC (0.09, 0.38, 0.25, 2.8, 19, 0.25), have lower False Acceptance Rates (FAR), False Negative Rates (FNR), False Disclosure Rates (FDR), False Positive Rates (FPR), Mathew Correlation Coefficients (MCC), and False Rejection Rates (FRR).

**Figure 9:**
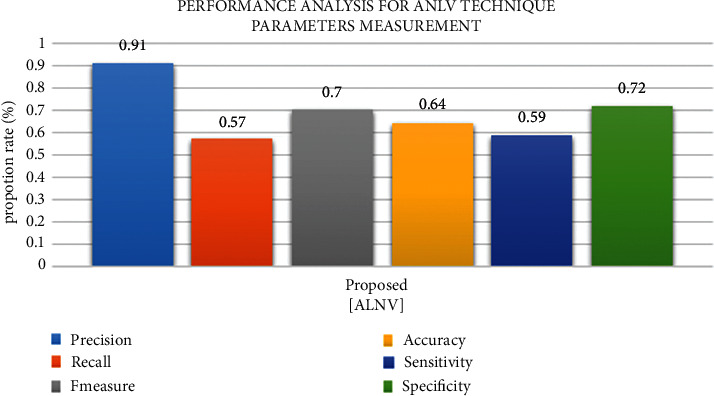
Comparison graph of dataset 1.

Accuracy results are shown in Figure 9 as estimates of the following: recall, F-measure, accuracy, specificity, and sensitivity. These values were produced using an LNNC-based EEG level estimation.

**Figure 10:**
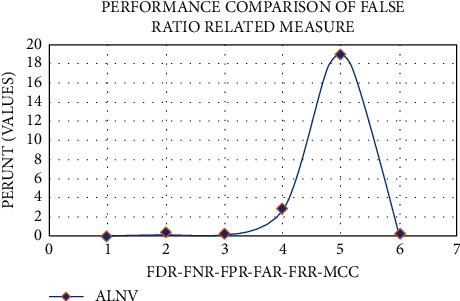
Comparison graph of dataset 1.

Figure 10 depicts that the FDR, FNR, FPR, FAR, FRR, and MCC estimations have been somewhat lowered, which have been utilised to increase the accuracy measurements of the EEG level estimate.

## 5. Conclusion

In order to distinguish between brain activity estimate and nerve skin of stimuli, advancements in laptop invention, and motorized knowledge licenses integrated into anthropological brains with produced gadgets, these forms of interfaces have been designated as Brain-Computer Interfaces (BCI). The BCI is not a framework that must be cast off to send productions and instructions to the outside world in conventional ways; rather, it is a caring of communication outline that recognizes and disrupts miserable intelligence activity and communicates it to the outdoor creation in conventional ways. A few parameters are examined in light of the new approaches that have been presented. Depending on the technique used, outline assortments show a distinct perspective. A set of incentive criteria must be constructed in order to conduct surveys to examine these aspects and improve estimates of enforcement rules. With the distinctive features of sleepy collecting rules, two metrics, and assurance that the suggested EEG state estimate strategy is more efficient than both approaches, the proposed LNNC methods may be compared and contrasted. The proposed work's outcomes are 0.91, 0.57, 0.70, 0.64, 0.59, and 0.72, demonstrating that the proposed frameworks, for example, LNNC, provide better results. According to the results of the examination in Table 2, the FDR, FNR, FPR, FAR, FRR, and MCC of the suggested systems, for example, LNNC, are 0.09, 0.38, 0.25, 2.8, 19, and 0.25, demonstrating that the new LNNC method has a lower false ratio than the existing LNNC approach.

## Figures and Tables

**Table 1:** Input datasets.

Category of database	Input samples	Input bandwidth	Overall sampling (KHz)	Data structure	Length of data
Sleep of the child	50	0.001 Hz–50 Hz	1.2	32 bits	8 minutes
Abnormal rate of the child from PhysioNet database	50	0.001 Hz–50 Hz	1.0	8 bits	7 minutes

**Table 2:** Comparison table of dataset 1.

Performance metrics	Methodology proposed
Precision	0.91
Recall	0.57
F-measure	0.70
Accuracy	0.64
Sensitivity	0.59
Specificity	0.72
FDR	0.09
FNR	0.38
FAR	2.8
FRR	19
MCC	0.25

## Data Availability

The data that support the findings of this study are available upon request from the corresponding author.
